# Facets of the Rift Valley Fever Outbreak in Northeastern Province, Kenya, 2006–2007

**DOI:** 10.4269/ajtmh.2010.09-0800

**Published:** 2010-03

**Authors:** Charles H. King, Summerpal S. Kahlon, Samuel Muiruri, A. Desiree LaBeaud

**Affiliations:** Center for Global Health and Diseases, CWRU School of Medicine, Cleveland, Ohio; Department of Medicine, University of Texas Medical Branch, Galveston, Texas; Division of Vector Borne and Neglected Tropical Diseases, Ministry of Public Health and Sanitation, Nairobi, Kenya; Children's Hospital Oakland Research Institute, Oakland, California

Rift Valley fever virus (RVFV) is a mosquito-borne *Phlebovirus* that causes periodic outbreaks of animal and human disease in Africa and the Arabian Peninsula. On the basis of its many competent vectors, its potential for aerosol transmission, and its progressive spread from East Africa to neighboring regions between 1950 and 2000, RVFV is ranked as a high-priority, emerging health threat for humans, livestock, and wildlife in all parts of the world.

Rift Valley fever virus is typically maintained by vertical transmission among floodwater *Aedes* species. Most often, local virus propagation is reactivated as these mosquitoes emerge from temporary ponds (dambos) formed by heavy rainfall in enzootic/endemic areas.^1^ Successive mosquito breeding near amplifying domestic livestock (cattle, goats, or sheep) allows for local intensification of exposure by bridge vectors such as *Culex*. Because livestock miscarriage and mortality rates are high, humans can also become occupationally exposed to RVFV by handling infected animal tissues or by aerosolization of body fluids.^2^ Human RVFV infection is almost always symptomatic (see Kahlon and others, this issue), typically presenting as a syndrome of fever with nausea and arthralgias, sometimes progressing to meningoencephalitis (10%), uveitis/retinitis (10–30%), or to a hemorrhagic diathesis (1%) that is highly lethal. Combined human disease and livestock losses are frequently devastating to affected communities.

**Figure 1.**
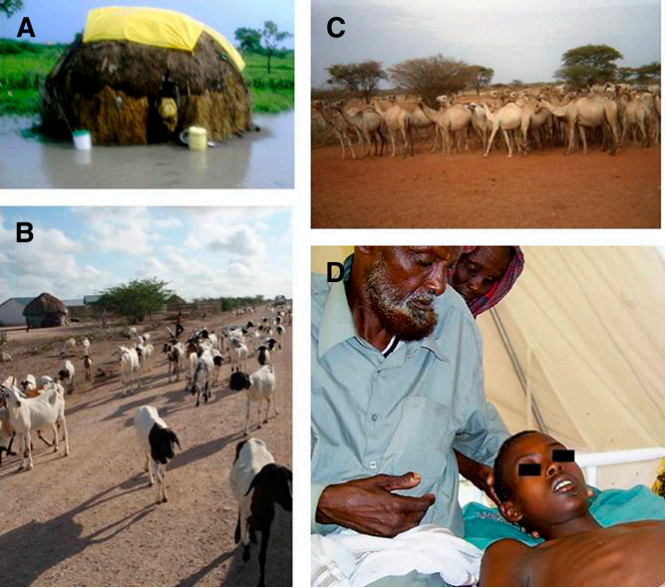
Panel A, persistent flooding from heavy rains; Panels B and C, susceptible livestock near study site; Panel D, local resident manifesting severe meningismus during encephalitic phase of confirmed RVF. (Photo credits: A, Samuel Muiruri; B, Desirée LaBeaud; C, Charles King; D, Summerpal Kahlon). This figure appears in color at www.ajtmh.org.

Figure 1A shows persistent local flooding associated with high numbers of peri-domestic vector mosquitoes in Ijara District, NE Province, following anomalous heavy rains linked with the El-Nino/Southern Oscillation event in 2006.^1^ Figure 1B and C show local abundance of livestock capable of amplifying Rift Valley fever virus transmission within semi-nomadic pastoralist communities.^2^ Figure 1D shows severe meningismus in a patient with fever and meningoencephalitis in January 2007, later confirmed to have acute Rift Valley fever virus infection.
